# Androgen deprivation promotes diabetic wound healing in mice through modulation of wound microbiome and immune response

**DOI:** 10.3389/fmicb.2025.1684165

**Published:** 2025-10-24

**Authors:** Ziyang Sun, Rizhong Huang, Jingyi Chen, Yangyu Song, Zihao Sun, Lixiang Zhang, Huaikai Shi, Ruoyu Mu, Yiwei Wang, Jinlong Huang, Xin Yan, Qian Tan

**Affiliations:** ^1^Department of Burns and Plastic Surgery, Nanjing Drum Tower Hospital, Clinical College, Nanjing University of Chinese Medicine, Nanjing, China; ^2^School of Pharmacy, Nanjing University of Chinese Medicine, Nanjing, China; ^3^Jiangxi Business Technology Institute, Nanchang, Jiangxi, China; ^4^Business Intelligence & Data Analytics (BIDA) Program, Heinz College, Carnegie Mellon University, Pittsburgh, PA, United States; ^5^Asbestos and Dust Diseases Research Institute, Concord, NSW, Australia; ^6^Department of Plastic Surgery, Affiliated Hospital of Nanjing University of Chinese Medicine, Nanjing, Jiangsu, China; ^7^Department of Burns and Plastic Surgery, Nanjing Drum Tower Hospital, Affiliated Hospital of Medical School, Nanjing University, Nanjing, China

**Keywords:** diabetic wounds, wound microbiome, androgen deprivation, 16S rRNA sequencing, inflammation

## Abstract

**Introduction:**

Delayed wound healing is a major complication of diabetes, often associated with chronic inflammation and microbial dysbiosis. Although androgens are known to impair wound repair, their role in diabetic wound healing, particularly in regulating the local wound microbiome and associated immune response, remains poorly understood. In this study, we investigated whether androgen deprivation via surgical castration could enhance diabetic wound healing by modulating local microbial communities and inflammation.

**Methods:**

A full-thickness wound model was established in db/db mice. Surgical castration was used to achieve androgen deprivation. Wound closure and histology were assessed longitudinally. Blood glucose and body weight were monitored. The local immune microenvironment was profiled, focusing on pro-inflammatory factors and macrophage polarization. 16S rRNA sequencing characterized α-diversity and community composition over time. Functional prediction analyses inferred microbial metabolic potential, and machine-learning models evaluated taxa associated with healing dynamics.

**Results:**

Androgen deprivation significantly accelerated wound closure and improved histological outcomes without altering blood glucose or body weight. The wound microenvironment showed reduced pro-inflammatory factors and enhanced M2 macrophage polarization. 16S rRNA sequencing revealed increased microbial α-diversity and durable shifts in community composition, most prominently during early healing. *Escherichia-Shigella, Rhodococcus*, and *Ochrobactrum* were enriched, while *Staphylococcus abundance* decreased. Functional prediction indicated elevated microbial metabolic activity after castration. Machine-learning analysis identified *Escherichia-Shigella* as a key genus associated with accelerated healing.

**Discussion:**

Low androgen levels were associated with improved diabetic wound repair, potentially by attenuating local inflammation and fostering a more diverse, metabolically active microbiota. These data support a mechanistic link among androgens, wound inflammation, and the microbiome, and suggest host-directed therapeutic strategies for chronic diabetic wounds.

## 1 Introduction

Diabetes has emerged as a major global public health challenge. According to the International Diabetes Federation, the number of individuals with diabetes in China reached 114 million by 2019, ranking the highest in the world (Liu et al., 2023). Among many complications of diabetes, chronic diabetic foot ulcers (DFUs) are particularly common and difficult to manage, primarily due to persistent hyperglycemia and impaired wound healing (Armstrong et al., 2023; Wang et al., 2024). Current treatments for DFUs rely heavily on antibiotics, debridement, and wound dressings (Wang et al., 2024), but the outcomes remain poor. Once a DFU develops, the 5-years cumulative mortality rate has been reported as 50%–70% (Armstrong et al., 2023). Under physiological conditions, wound healing proceeds through four overlapping and tightly regulated phases: hemostasis, inflammation, proliferation, and remodeling, mediated by intricate cell-to-cell and cell–extracellular matrix interactions (Eming et al., 2014; Peña and Martin, 2024). However, in the diabetic condition, chronic hyperglycemia and oxidative stress disrupt this process, leading to prolonged inflammation, delayed clearance of neutrophils and macrophages, poor angiogenesis, reduced fibroblast and keratinocyte proliferation, and impaired re-epithelialization (Huang et al., 2025; Patel et al., 2019; Xiong et al., 2025). Consequently, there is an urgent need to better understand the molecular and ecological mechanisms that regulate diabetic wound repair to reduce the incidence and mortality associated with DFU-related complications.

Recent studies have demonstrated that modulation of the wound microbiome can significantly enhance diabetic wound healing (Xu et al., 2025). Particularly, increased microbial diversity has been shown to suppress pathogen overgrowth and promote tissue repair (Naik et al., 2012). In diabetic wounds, *Staphylococcus* spp. Are the most commonly identified microorganisms, and current clinical treatments often aim to reduce or eliminate *Staphylococcus* colonization using antibiotics (Macdonald et al., 2021). However, one study reported that broad-spectrum antibiotic treatment, while effective in reducing microbial load, also depleted beneficial commensal bacteria, ultimately delaying wound closure in diabetic ulcers (Khadka et al., 2024). These findings highlight the limitations of conventional antibiotic therapies and underscore the importance of preserving or restoring a balanced wound microbiome to support effective healing (Wang et al., 2021).

Testosterone is the primary androgen in both men and male mice (Lawrence et al., 2022). While it is predominantly produced by the testes, testosterone can also be synthesized from androgen precursors in peripheral tissues such as the liver, skin, adipose tissue, and prostate (Barsky and Monks, 2025). Androgens have been shown to influence the composition of skin bacterial and fungal communities, particularly during puberty (Park et al., 2022). Furthermore, the slower wound healing observed in elderly men suggests that androgens play a regulatory role in tissue repair (Ashcroft and Mills, 2002; Shi et al., 2021). Beyond metabolic and microbial factors, androgen signaling constitutes a critical endocrine axis involved in wound healing (Becerra-Díaz et al., 2018; Shi et al., 2020). Androgens such as testosterone have been shown not only to shape secondary sexual characteristics but also to modulate immune responses and tissue regeneration (Jeschke et al., 2007; Lai et al., 2009). Animal studies demonstrated that surgical castration or pharmacological blockade of the androgen receptor (AR) using agents such as flutamide significantly accelerates wound closure and reduces inflammation (Ashcroft and Mills, 2002; Romana-Souza et al., 2014; Toraldo et al., 2012). In contrast, elevated systemic androgen levels have been associated with delayed acute wound healing (Ashcroft and Mills, 2002; Shi et al., 2021). Mechanistically, AR signaling in keratinocytes and macrophages promotes TNF-α expression, inhibits the β-catenin/TGF-β signaling pathway, and suppresses M2 macrophage polarization, collectively hindering re-epithelialization and the resolution of inflammation (Gilliver et al., 2009; Lai et al., 2009). Moreover, castration has been shown to restore the dendritic cell (DC)–type 2 innate lymphoid cell (ILC2) axis, enhance cutaneous immunity, reduce pathogen burden, and increase microbial diversity in the skin (Chi et al., 2024). These findings suggest a complex interplay between androgen signaling, wound microbiome, and immune regulation. However, the precise role of the wound microbiome in this context remains unclear and has not been thoroughly investigated. In particular, the interactions among androgen signaling, local immune responses, and microbial communities within diabetic chronic wounds are largely unexplored, representing a gap in our understanding of how androgen modulation influences both the wound microbiome and immune response.

In this study, we aim to investigate whether androgen levels influence microbiome diversity during wound healing. Specifically, we will explore the role of androgen-mediated microbiome modulation in the wound repair process using a surgical castration model in *db*/*db* mice, combined with 16S rRNA sequencing and histological analyses. The findings from this study are expected to clarify the role of the androgen-microbiome–wound axis in regulating the healing process and to provide a novel conceptual and translational framework for developing advanced therapies for diabetic foot ulcers.

## 2 Materials and methods

### 2.1 Animals and experimental design

Male db/db mice (10 weeks old, weight: 40–50 g; *n* = 35 total; *n* = 5 per group at each time point: Day 3, 7, and 14) were purchased from Jiangsu GemPharmatech Co., Ltd. All animals were housed in specific pathogen-free (SPF) animal facility at the Nanjing University of Chinese Medicine. The environment was controlled temperature of 24 °C–26 °C, relative humidity of 44%–46%, under a 12-h light/dark cycle with lights on at 6 am. All protocols were approved by the Animal Ethic Committee, Nanjing University of Chinese Medicine (Approval Number: 202505A063) and in accordance with the Guidelines of the Ministry of Science and Technology of the People’s Republic of China (MOST, 2006) for the Care and Use of Laboratory Animals.

### 2.2 Castration procedure and surgical wounding

At 10 weeks of age, mice were randomly assigned to either the control group or the castration group. Mice were anesthetized with 3% isoflurane prior to orchiectomy under sterile conditions. The scrotal area was shaved and thoroughly disinfected with povidone-iodine. Bilateral incisions (∼5 mm) were made on each side of the scrotum to individually expose the testes and spermatic cords. The spermatic cords were ligated using absorbable sutures, and the testes were excised. After confirming hemostasis, the scrotal incisions were closed with absorbable sutures. Postoperative analgesia was provided by subcutaneous administration of buprenorphine (0.05 mg/kg) immediately following surgery and repeated every 24 h for up to 48 h. Mice were placed on a heating pad postoperatively until fully recovered. Post-surgery, animals were housed individually and fed chow diet *ad libitum* for 7 days prior to surgical wounding (Shi et al., 2020, 2022).

To create wound injury, all animals were anesthetized with 3% isoflurane and the dorsum of each mouse was shaved. An excisional wound at 1 cm^2^ was created surgically on the dorsal skin. Wounds were covered with non-adherent Atrauman^®^ (HARTMANN, Heidenheim, China) as the primary dressing, then secured with IV3000 (Smith & Nephew, China). Finally, a 2–3 cm length of cylindrical Flexinet (Winner^®^, China), a compressive elastic net dressing, was applied over the dressings. All three layers were secured by passing three dorsal sutures of 5-o silk (Johnson & Johnson, USA) through the dressing layers and skin. Post wounding, animals were placed on a heating pad postoperatively until fully recovered. Mice were housed individually and fed chow diet *ad libitum*. Analgesia (intraperitoneal carprofen 5 mg/kg) was provided daily for 4 days after wounding. Mice were monitored daily for the first 10 days for any signs of distress, measuring body weight and assessing any changes in physical appearance and behavior (grooming, shivering, activity, skin wounds). No adverse events such as wound infection were observed. Digital photographs of the wound area were captured on Days 0, 3, 7, and 14 post-injuries using a standardized imaging system under consistent lighting and distance conditions. Wound size was quantified using ImageJ software (version 2.1.0/1.53c).

### 2.3 Tissue collection and processing

On Days 3, 7, 14 post injuries, wound tissues were harvested and collected for histological and molecular analysis. Blood was collected via retro-orbital bleeding, and serum was separated by centrifugation at 10,000 rpm for 5 min and stored at −80 °C for hormone analysis. After tissue collection, all animals were euthanized immediately by cervical dislocation.

### 2.4 Histological and immunohistochemistry

All wound tissues were fixed in 10% neutral-buffered formalin for 24 h at room temperature, followed by dehydration in a graded ethanol series, clearance in xylene, and paraffin embedding. Paraffin-embedded blocks were sectioned at a thickness of 5 μm, dewaxed, and rehydrated. Sections were subjected to hematoxylin and eosin (H&E) staining for general histological evaluation and Masson’s trichrome staining for collagen deposition. Histological features, including re-epithelialization and inflammatory cell infiltration, were examined using a light microscope (Axio Vert A1, ZEISS). Collagen fiber density was quantified using ImageJ software (version 2.1.0/1.53c).

Immunohistochemical staining for CD86 and CD206 was performed on 4 μm paraffin-embedded wound tissue sections. After deparaffinization, rehydration, and heat-induced antigen retrieval in citrate buffer (pH 6.0), endogenous peroxidase was quenched with 3% H_2_O_2_ and sections blocked with 5% BSA. Slides were incubated overnight at 4 °C with primary antibodies: anti-CD206 (CST, Cat. No. 24595, Lot 2024FA0520) at 1:800 and anti-CD86 (CST, Cat. No. 19589S, Lot 2024FA0523) at 1:200. After incubation with HRP-conjugated secondary antibody and DAB development, hematoxylin counterstaining was applied. CD206 positivity was defined by brown cytoplasmic/membrane staining, and CD86 by brown membrane staining. Positive cells were quantified by two independent observers using Fiji ImageJ software (version 2.1.0/1.53c).

### 2.5 Serum hormone quantification by LC-MS/MS

Serum levels of testosterone (T) and dihydrotestosterone (DHT) were quantified using liquid chromatography–tandem mass spectrometry (LC–MS/MS) with isotopically labeled internal standards as previously established in our lab (Shi et al., 2020, 2022). Briefly, 100 μL of mouse serum was mixed with deuterated testosterone (testosterone-D3, CDVD-A201-1005, Anpel) and deuterated dihydrotestosterone (5α-dihydrotestosterone-D3, D-077-1ML, Sigma-Aldrich) as internal standards. After vertexing, proteins were precipitated with cold methanol, and the supernatant was collected and filtered using a 0.22 μm PTFE syringe filter (B1100-dfk, disposable type).

Chromatographic separation was performed on a C18 reversed-phase column under gradient elution conditions. The analysis was conducted using a Thermo Scientific TSQ triple quadrupole mass spectrometer equipped with an electrospray ionization (ESI) source operating in positive ion mode. Multiple reaction monitoring (MRM) transitions specific for testosterone and DHT were used for detection. Quantification was based on the peak area ratios of the target analytes to the internal standards. Data acquisition and processing were performed using the instrument’s proprietary software.

### 2.6 RNA isolation and quantitative real-time polymerase chain reaction

The wound tissues collected from the diabetic wound on Days 3, 7 and 14 post-injury were mechanically homogenized and used for RNA extraction. mRNA from wound tissues was extracted using Trizol reagent (Invitrogen, Carlsbad, CA, USA). Total RNA (1 μg) was reverse-transcribed to complementary DNA using the SensiFAST cDNA synthesis kit (Bioline, London, US). Real-time PCR analysis was then conducted using SsoAdvanced Universal SYBR Green Supermix (Bio-Rad, Hercules, CA, USA). The efficiency of DNA amplification was evaluated using the mean cycle threshold (*C*_t_) method. ΔCt value was calculated from *C*_t_ values of different interest genes by subtracting the *C*_t_ value of the housekeeping gene, β-actin. The relative mRNA expression was shown as fold change (2^(−ΔΔCt)) relative to the expression in baseline. The primer sequence (Sangon Biotech, Shanghai, China) is set as in Table 1.

**TABLE 1:** Sequences of the primers designed for RT-PCR.

Gene	Forward primer	Reverse primer
*Il-1*β	GAAATGCCACCTTTTGACAGTG	TGGATGCTCTCATCAGGACAG
*Il-10*	CAAACAGTACGGAAACTCAACCT	GGTGATACAGATCCAGGGTGAAC
*Mrc1*	GTGGGGACCTGGCAAGTATC	CACTGGGGTTCCATCACTCC
*Il-6*	GTCCTTCCTACCCCAATTTCCA	TAACGCACTAGGTTTGCCGA
*Act*β	GGCTGTATTCCCCTCCATCG	CCAGTTGGTAACAATGCCATGT

### 2.7 16S rRNA sequencing and microbiota analysis

Microbial community profiling was performed by Meiji Biosciences (China). Total genomic DNA was extracted from samples using the QIAamp DNA Stool Mini Kit (Qiagen, Germany) according to the manufacturer’s instructions. The V3–V4 hypervariable regions of the bacterial 16S rRNA gene were amplified using primers 341F (5′-CCTACGGGNGGCWGCAG-3′) and 806R (5′-GGACTACHVGGGTATCTAAT-3′). Amplicons were purified, quantified, and sequenced on the Illumina MiSeq platform (2 × 250 bp paired-end reads). Raw reads were processed using QIIME2 (version 2021.4), including quality control, denoising with DADA2, and chimera removal. Taxonomic assignments were performed based on the SILVA 138 reference database.

Statistical analysis and data visualization were conducted in R. Alpha diversity was assessed using Chao, ACE, Sobs, and Shannon indices, and beta diversity was visualized through principal coordinate analysis (PCoA) based on Bray–Curtis distances. Additional visualizations included bar plots at phylum and genus levels, Venn diagrams, and Circos plots. Differential abundance analyses were performed using both pairwise statistical tests and Linear Discriminant Analysis Effect Size (LEfSe). Functional prediction was then performed using Phylogenetic Investigation of Communities by Reconstruction of Unobserved States (PICRUSt2) to generate Kyoto Encyclopedia of Genes and Genomes (KEGG) and Clusters of Orthologous Groups (COG) based heatmaps. Raw 16S rRNA sequencing reads have been deposited to the NCBI Sequence Read Archive under BioProject PRJNA1305122.

### 2.8 AI-based random forest modeling with SHAP interpretation

Microbiome-based predictive modeling was established in Python using the scikit-learn ecosystem. After centered log-ratio transformation and standard scaling of the genus-level abundance data, a random-forest algorithm with stratified cross-validation and grid-based hyper-parameter optimization was trained to discriminate healing versus non-healing wounds. Model-agnostic interpretation was performed with SHapley Additive exPlanations (SHAP). For each sample, SHAP values were computed to quantify the marginal contribution of every genus to the predicted probability, and population-level importance was summarized as the mean absolute SHAP value across all observations. Genera with the highest SHAP impact were considered key microbial determinants of wound prognosis and were subjected to downstream pathway enrichment analysis. All analyses were executed on a workstation running Python 3.10, and the workflow adhered to best-practice guidelines for reproducible machine-learning in biomedical research.

### 2.9 Statistical analysis

All data are presented as the mean ± standard deviation (SD). Statistical analyses were performed using GraphPad Prism version 9.0 (GraphPad Software, USA). Two-group comparisons were conducted using unpaired Student’s *t*-test, while multifactorial comparisons across groups and timepoints were evaluated using two-way ANOVA followed by Tukey’s multiple comparisons test. *P*-values < 0.05 were considered statistically significant.

## 3 Results

### 3.1 Castration accelerates wound healing in diabetic male mice

**FIGURE 1:**
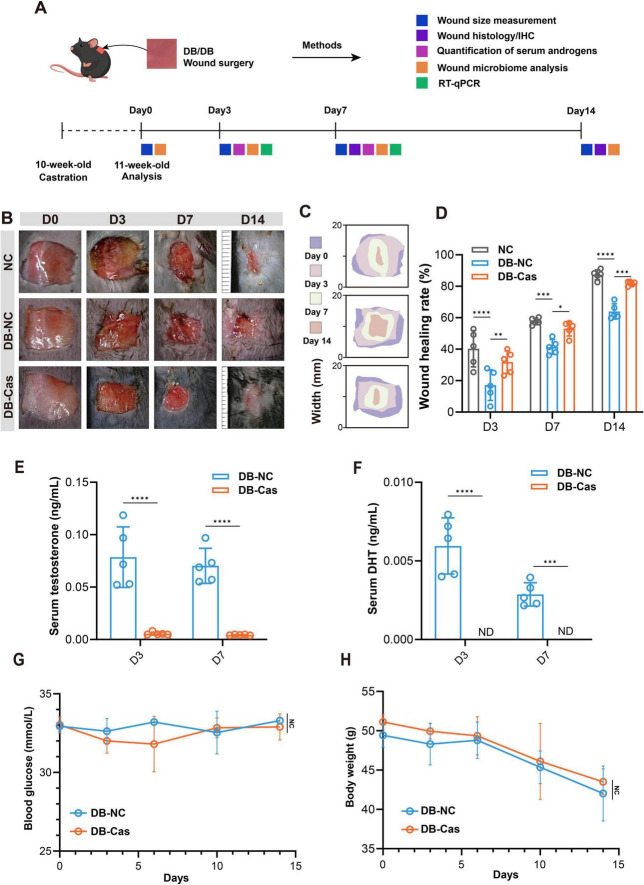
Castration accelerates wound healing in diabetic mice by reducing serum androgen levels. **(A)** Experimental scheme. **(B)** Representative wound images from NC (normal control), DB-NC (diabetic non-castrated), and DB-Cas (diabetic castrated) groups at Days 0, 3, 7, and 14 post-injury. **(C)** Heat map of wound closure over time. **(D)** Quantitative analysis of wound healing rates at Days 3, 7, and 14. **(E)** Serum levels of testosterone and **(F)** dihydrotestosterone (DHT) in DB-NC and DB-Cas on Days 3 and 7. **(G,H)** Longitudinal measurements of blood glucose **(G)** and body weight **(H)** over 14 days. Data are presented as mean ± SD. *n* = 5 mice per group. Statistical significance was determined using unpaired two-tailed *t*-tests or two-way ANOVA, as appropriate. *p < 0.05, **p < 0.01, ***p < 0.001, ****p < 0.0001.

Androgens are reported to have a contradictory role in wound healing (Shi et al., 2021). Here, we examined whether castration could reverse impaired wound repair in diabetic mice (Figure 1A). Diabetic castrated male mice (DB-Cas) exhibited accelerated healing at all timepoints (Figures 1B–D). By Day 3, DB-Cas wounds healed 31.8% ± 7.2%, nearly double the closure in non-castrated diabetic mice (DB-NC, 17.1% ± 9.8%) and approaching wild-type controls (NC; 40.3% ± 11.5%). The advantage persisted on Day 7 (DB-Cas: 53.0% ± 4.6% vs. DB-NC: 41.4% ± 4.7%) and Day 14 (DB-Cas: 81.8% ± 1.4% vs. DB-NC: 64.0% ± 4.9%), with DB-Cas nearing NC performance (87.3% ± 3.1%). These results indicate that androgen ablation robustly enhanced diabetic wound repair, aligning it closely with wild-type healing (Figures 1C, D). LC-MS/MS analysis confirmed successful castration, with serum testosterone (T) and dihydrotestosterone (DHT) levels in DB-Cas mice reduced to baseline or undetectable levels on Days 3 and 7 (Figures 1E, F). Additionally, castration did not alter systemic metabolism: blood glucose and body weight remained unchanged between DB-Cas and DB-NC mice throughout the study (Figures 1G, H). These results demonstrate that androgen depletion accelerates diabetic wound healing largely independently of metabolic effects.

### 3.2 The effect of androgen deprivation on inflammation over the healing process

**FIGURE 2:**
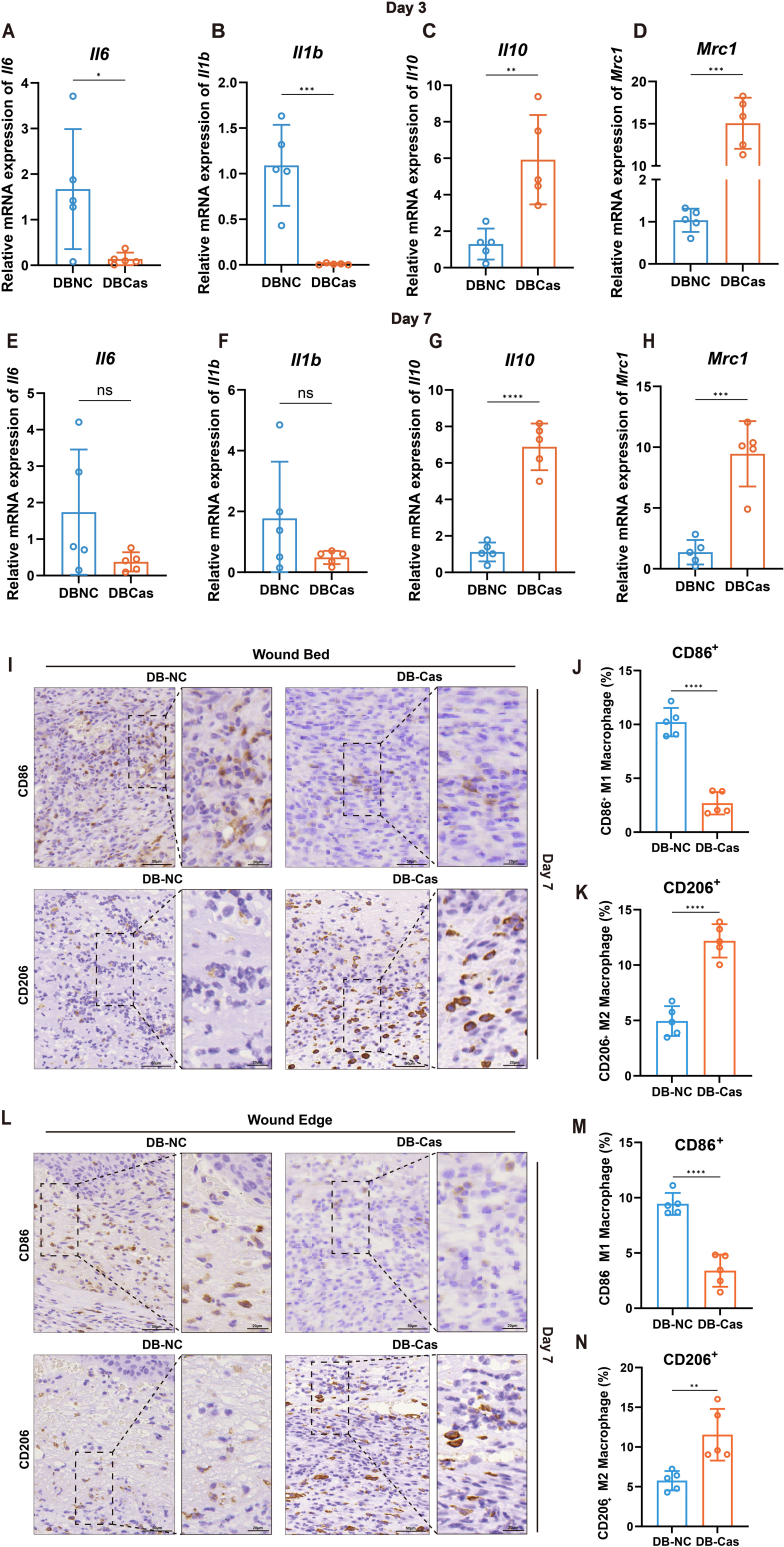
Wound macrophage phenotype and cytokine expression. **(A–D)** RT-qPCR analysis of *Il6*, *Il1b*, *Il10*, and *Mrc1* expression in wound tissues on Day 3. **(E–H)** RT-qPCR analysis of the same cytokines and markers on Day 7. *n* = 5 mice per group. Data are shown as mean ± SD. **(I)** IHC staining of wound bed at Day 7. **(J,K)** Quantification of CD86^+^ and CD206^+^ macrophages, expressed as a percentage of total cells at the wound bed. **(L)** IHC staining of wound edge on Day 7. **(M,N)** Quantification of CD86^+^ and CD206^+^ macrophages, expressed as a percentage of total cells at the wound edge. *n* = 5 mice per group. Two-tailed unpaired *t*-test; *p < 0.05, **p < 0.01, ***p < 0.001, ****p < 0.0001.

We first performed RT–qPCR analysis on wound tissues at Days 3 and 7 to characterize transcriptional changes in inflammatory mediators. At Day 3, the expression of pro-inflammatory cytokines *Il6* and *Il1b* was significantly downregulated in DB-Cas compared to DB-NC (*p* < 0.05 and *p* < 0.001, respectively; Figures 2A, B), indicating suppressed early inflammatory signaling. Moreover, transcripts encoding the anti-inflammatory cytokine *Il10* and the M2 macrophage marker *Mrc1* were markedly elevated in DB-Cas wounds (*p* < 0.01 and *p* < 0.001, respectively; Figures 2C, D), suggesting enhanced M2-like macrophage activity. By Day 7, the expression levels of *Il6* and *Il1b* no longer differed significantly between groups (Figures 2E, F). However, both cytokines exhibited a downward trend in the DB-Cas group, suggesting a continued suppression of pro-inflammatory signaling despite the absence of statistical significance. In contrast, *Il10* expression remained markedly elevated in DB-Cas wounds (*p* < 0.0001; Figure 2G), and *Mrc1* expression also persisted at significantly higher levels (*p* < 0.001; Figure 2H). These sustained transcriptional alterations were consistent with immunohistochemical (IHC) findings showing an increased presence of CD206^+^ (M2) macrophages and a reduction in CD86^+^ (M1) macrophages, indicating a prolonged shift toward a reparative immune phenotype. Subsequently, we conducted IHC and analyses on wound tissue sections collected at Day 7 post-injury (Figures 2I–N), assessing macrophage polarization markers. In the DB-Cas group, both in the wound bed and wound edge, there was a pronounced decrease in CD86^+^ (M1-like) macrophages and a corresponding increase in CD206^+^ (M2-like) macrophages, relative to DB-NC (Figures 2I, L). Quantitative analysis confirmed a robust shift toward an anti-inflammatory M2 macrophage phenotype (Figures 2K, N) and a reduction in the M1 macrophage phenotype (Figures 2J, M).

Collectively, these findings demonstrate that androgen deprivation attenuates early pro-inflammatory gene expression and promotes an immune microenvironment that favors M2 macrophage polarization, thereby facilitating diabetic wound repair.

### 3.3 Androgen deprivation accelerated re-epithelization, collagen deposition, and cell proliferation

**FIGURE 3:**
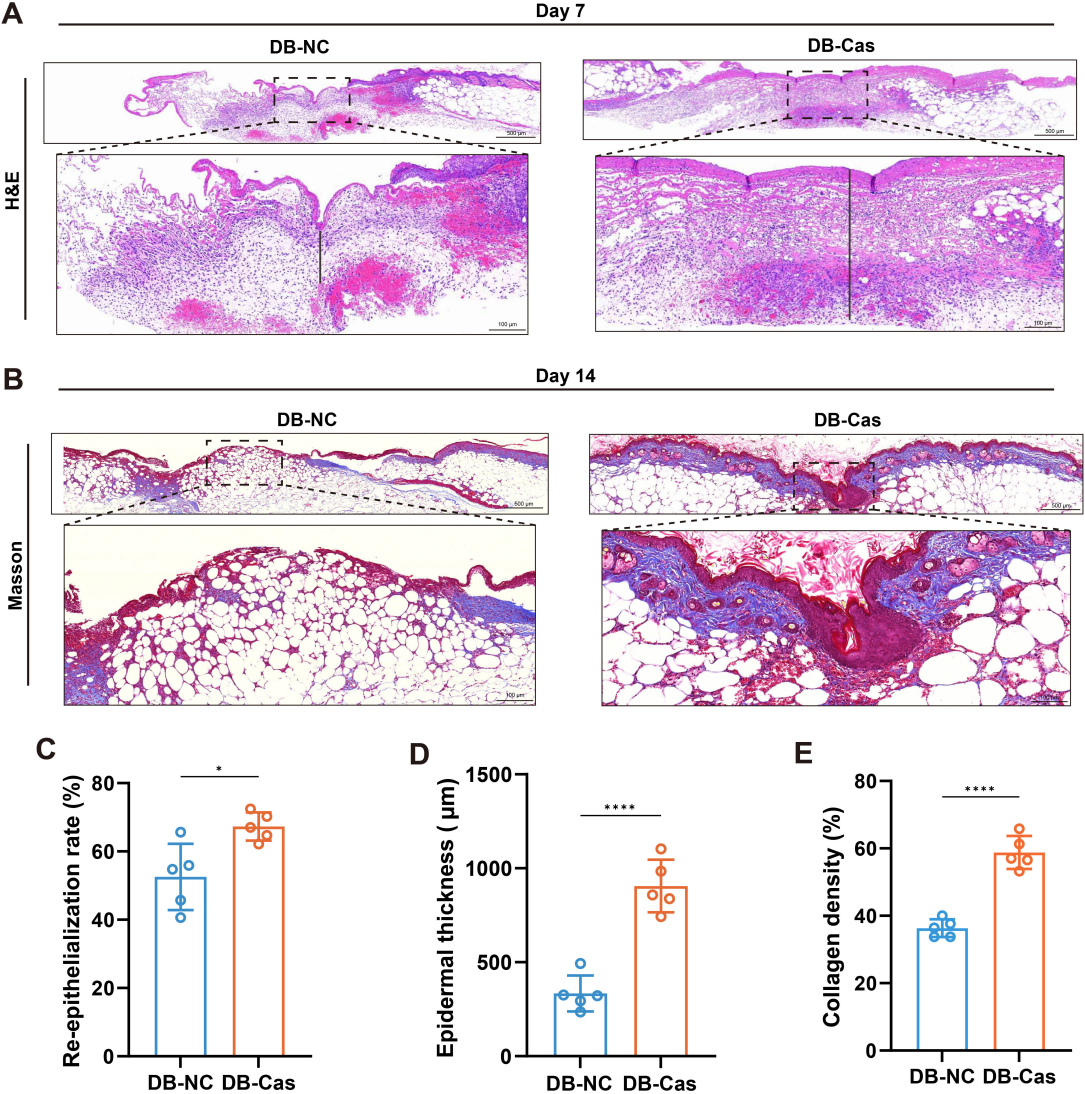
Androgen deprivation enhances re-epithelialization and collagen deposition in diabetic wounds. **(A,B)** Representative histological images of wound tissue from DB-NC and DB-Cas on Day 7, stained with **(A)** hematoxylin and eosin (H&E) and **(B)** Masson’s trichrome. Dashed lines indicate wound margins. Scale bars: 500 μm (upper panels) and 100 μm (lower panels). **(C)** Quantification of the re-epithelialization rate based on H&E staining. **(D)** Quantification of newly formed epidermal thickness. **(E)** Quantification of collagen volume fraction based on Masson’s trichrome staining. Data are presented as mean ± SD. *n* = 5 mice per group. Statistical significance was determined using unpaired two-tailed *t*-tests. *p < 0.05, ****p < 0.0001.

Histological analysis on Day 7 post-wounding revealed that wounds in the DB-Cas group exhibited markedly improved tissue regeneration compared to DB-NC (Figure 3A). H&E staining showed enhanced re-epithelization and a thicker neo-epidermis in the DB-Cas group (Figure 3A). Quantitative analysis confirmed a significant increase in the re-epithelialization rate (Figure 3C, *p* < 0.05) and epidermal thickness (Figure 3D, *p* < 0.0001) in castrated mice. On Day 14, Masson’s trichrome staining further revealed greater collagen deposition in DB-Cas wounds (Figure 3B), indicating enhanced extracellular matrix remodeling. Consistently, the collagen density was significantly elevated in the DB-Cas group compared to controls (Figure 3E, *p* < 0.0001). Collectively, these findings suggest that androgen deprivation promotes more effective wound re-epithelialization and structural regeneration, at both the epithelial and matrix levels.

### 3.4 Androgen deprivation reshaped wound microbial community

**FIGURE 4:**
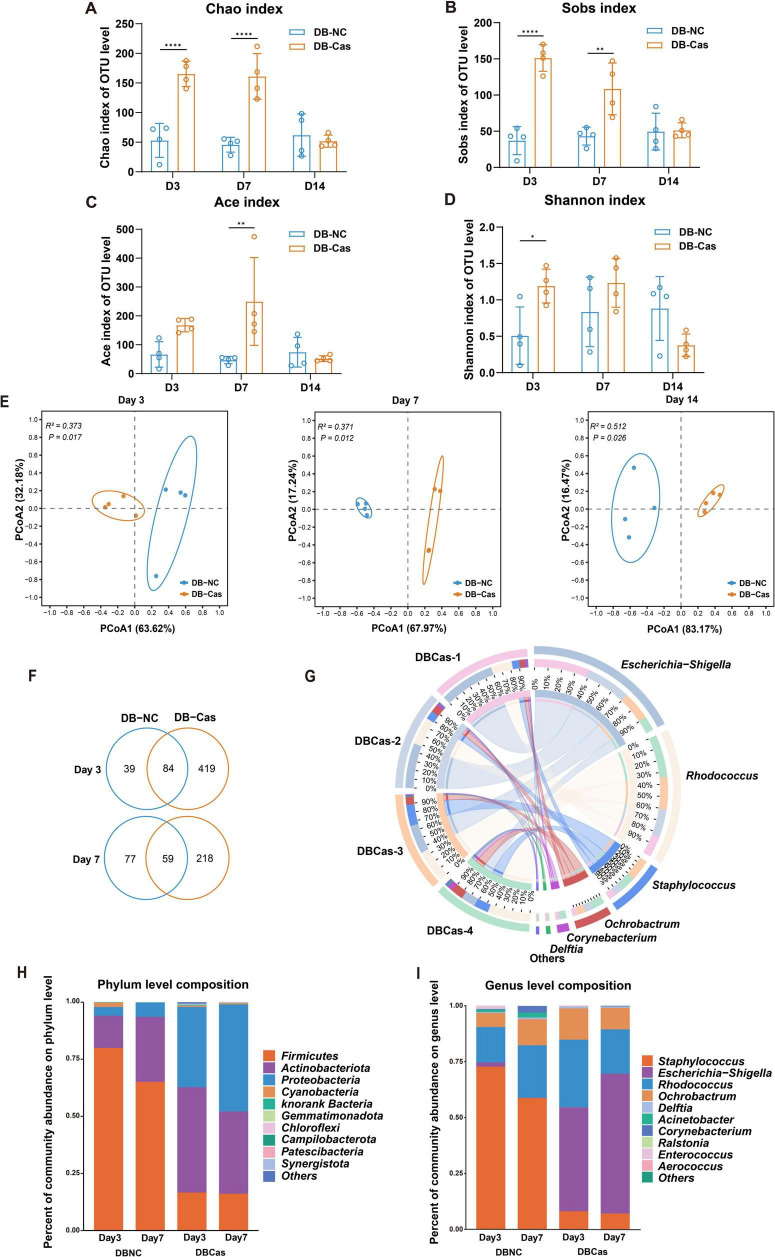
Analysis of wound microbiota composition and microbial diversity. **(A–D)** Alpha diversity indices at the OTU level, including Chao, Sobs, Ace, and Shannon, in DB-NC and DB-Cas groups at Days 3, 7, and 14; Significance was determined by Student’s *t*-test. **(E)** Principal coordinate analysis (PCoA) based on Bray–Curtis distances reveals distinct microbial community structures between DB-NC and DB-Cas at Days 3, 7, and 14; group separation at each time point was tested with PERMANOVA on Bray–Curtis distance matrices. **(F)** Circos plot illustrating the distribution and relative contributions of the most abundant genera on Day 7. **(G)** Venn diagrams showing the number of shared and unique OTUs between groups at Days 3 and 7. **(H)** Bar plot showing the bacterial community composition at the phylum level. **(I)** Genus-level composition showing enrichment of *Escherichia–Shigella*, *Rhodococcus*, and *Ochrobactrum* in the DB-Cas group, with reduced *Staphylococcus abundance*. *n* = 4 mice per group. Data are presented as mean ± SD. Significance was determined by Student’s *t*-test or PERMANOVA. *p < 0.05, **p < 0.01, ****p < 0.0001.

Castration significantly enhanced wound repair without affecting hyperglycemia, likely through modulation of the wound microbiome, as androgens are known to influence skin microbiota (Chi et al., 2024; Xu et al., 2025). To investigate this, we performed 16S rRNA sequencing on wound tissues collected at Days 3, 7, and 14 post-injury. DB-Cas wounds showed increased α-diversity, particularly during early healing (Days 3 and 7). On Day 3, castration elevated microbial diversity (∼3-fold higher Chao and Sobs indices, *p* < 0.0001) and Shannon index (*p* < 0.05), indicating enhanced richness and evenness. By Day 7, richness remained elevated. By Day 14, diversity indices converged, but early changes suggested sustained microbial restructuring (Figures 4A–D). Bray–Curtis PCoA revealed persistent compositional differences (Days 3/7/14: R^2^ = 0.373/0.371/0.512, *p* < 0.05; Figure 4E). Venn analysis showed DB-Cas wounds harbored more unique OTUs (Figure 4F). Circos analysis indicated a more diverse microbial network in castrated wounds, with increased interactions involving *Escherichia-Shigella, Rhodococcus, and Staphylococcus* (Figure 4G). Taxonomically, DB-Cas wounds exhibited reduced *Firmicutes* and enriched *Proteobacteria/Actinobacteria* (Figure 4H). At the genus level, *Staphylococcu*s declined, while *Escherichia-Shigella*, *Rhodococcus*, *Ochrobactrum*, and *Delftia* increased (Figure 4I). These findings demonstrate that androgen ablation remodels the wound microbiome toward greater diversity and potentially less pathogenic communities during critical healing phases.

### 3.5 Androgen deprivation enhances microbial metabolism and enriches *Escherichia-Shigella*

**FIGURE 5:**
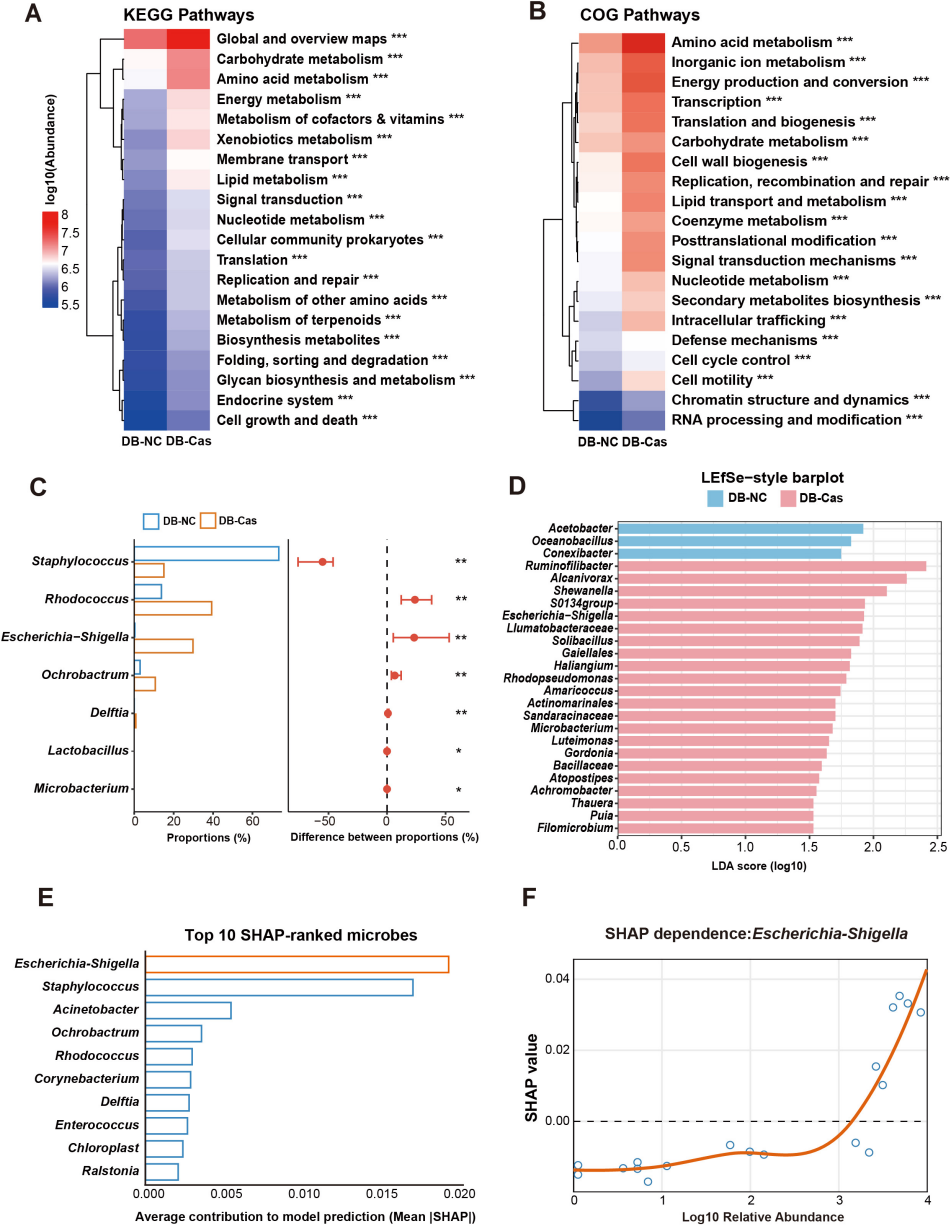
Functional prediction and machine learning identify *Escherichia–Shigella* as a key taxon associated with faster wound healing. **(A)** KEGG and **(B)** COG pathway enrichment heatmaps comparing DB-Cas and DB-NC on Day 7. Pathway differences were assessed with two-sided Welch’s *t*-tests. **(C)** Comparison of differentially abundant genera between DB-NC and DB-Cas groups on Day 7. Genus abundances were compared using two-sided Welch’s *t*-tests. **(D)** LEfSe analysis highlights taxa enriched in DB-Cas versus DB-NC, based on Kruskal-Wallis and Wilcoxon tests with effect size estimated by linear discriminant analysis, LDA score threshold = 2.0. **(E)** Random forest classification with SHAP identifies *Escherichia–Shigella* as the most important genus contributing to group discrimination. **(F)** SHAP dependence plot demonstrates that higher relative abundance of *Escherichia–Shigella* is positively associated with predictive contribution to wound healing in the model. *n* = 4 mice per group. Statistical significance: *p < 0.05, **p < 0.01, ***p < 0.001. Functional prediction based on Greengenes reference. LDA score threshold = 2.0.

To assess the functional impact of castration-induced microbiome changes, we performed KEGG and COG pathway analyses. These data revealed significant enrichment in metabolic pathways, including carbohydrate, amino acid, and lipid metabolism, energy metabolism, and signal transduction–in DB-Cas versus DB-NC wounds (Figures 5A, B). Comparative abundance analysis identified key genera associated with these shifts: *Microbacterium*, *Lactobacillus*, *Delftia*, *Ochrobactrum*, and *Escherichia-Shigella* were enriched in DB-Cas wounds, while *Staphylococcus* and *Rhodococcus* dominated DB-NC wounds (Figure 5C). LEfSe analysis confirmed these differential taxa, reinforcing their role in post-castration microbial divergence (Figure 5D). To identify taxa linked to healing improvement, we applied Random Forest modeling with SHAP analysis, pinpointing *Escherichia-Shigella* as the most influential genus (Figure 5E). SHAP dependence analysis further validated its predictive power, directly correlating higher *Escherichia-Shigella* abundance with enhanced healing outcomes (Figure 5F). Together, these results delineate a clear microbial-functional axis through which androgen depletion promotes diabetic wound repair.

## 4 Discussion

**FIGURE 6:**
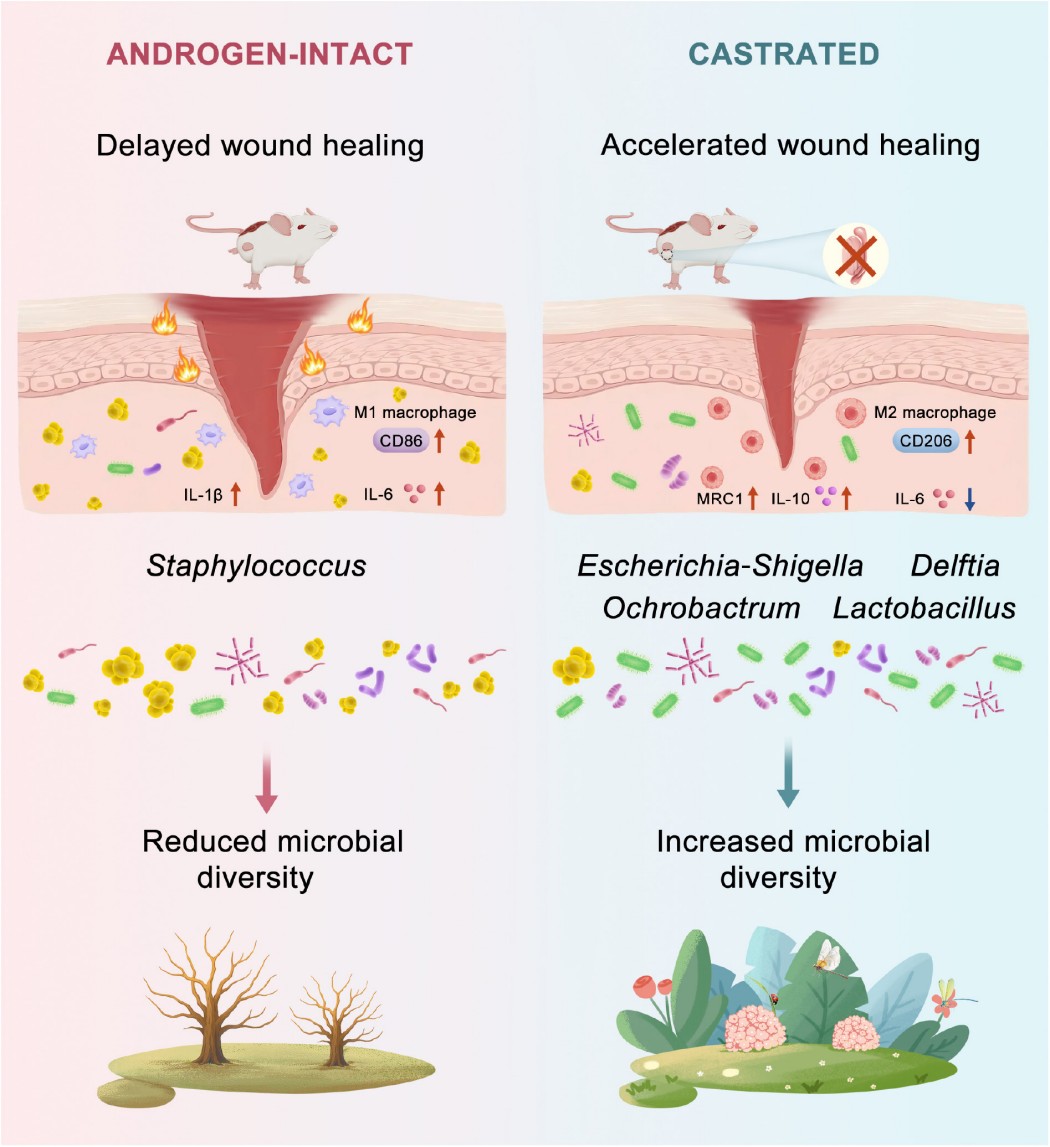
Proposed mechanism by which androgen deprivation promotes diabetic wound healing. Androgen deprivation modulates the wound microbiota by increasing microbial diversity and enriching beneficial taxa such as *Escherichia coli*. These microbial changes reduce pro-inflammatory cytokines (*Il-6*, *Il-1b*) and enhance anti-inflammatory mediators (*Il-10*, *Mrc1*), accompanied by a shift in macrophage polarization from M1 (CD86^+^) to M2 (CD206^+^) phenotypes. This coordinated immune–microbiota interaction accelerates re-epithelialization and collagen deposition, ultimately improving wound closure in diabetic mice.

In this study, we demonstrate that androgen deprivation through surgical castration significantly accelerates wound healing in a diabetic mouse model (Figure 6). Our results reveal that androgen deprivation positively influences diabetic wound repair, and this benefit is closely associated with microbiota reshaping at the wound site. Castrated db/db mice exhibited significantly accelerated wound healing. This improvement was attributed to reduced pro-inflammation, enhanced anti-inflammation and re-epithelialization, increased cell proliferation, and greater extracellular matrix production. These findings are partially consistent with earlier studies suggesting that androgens impair cutaneous wound repair by promoting TNF-α production (Lai et al., 2009). Interestingly, while exogenous DHT has been shown to exert anti-inflammatory effects and promote regeneration in severe burn models (Ashcroft and Mills, 2002; Shi et al., 2020). In our diabetic wound model, androgen deprivation appeared to promote an earlier transition from a pro-inflammatory to an anti-inflammatory phase during the early healing process. This apparent contradiction may stem from the differing microbial compositions between acute and diabetic wounds (Eisenstein, 2020) or various inflammatory responses (Shi et al., 2021). Moreover, in severe burn injury, systemic oxandrolone has been shown to enhance metabolism in children after injury and to dampen early inflammation, thereby accelerating wound closure (Murphy et al., 2004; Porro et al., 2012). By contrast, endogenous systemic androgens in adults tend to prolong inflammation following acute injury, whereas anti-androgens or AR blockade has been reported to enhance wound repair (Allam et al., 2023). In more complex situation, such as elderly individuals who exhibit both reduced androgen levels and metabolic rate, together with chronic inflammation state and diminished wound microbiome diversity (Li et al., 2023; Martin et al., 2024), localized androgen reduction may offer therapeutic benefit. Specifically, modulating androgen activity at the wound site could help reshape the wound microbiome and promote diabetic wound repair, while avoiding the adverse effects associated with systemic androgen deprivation. Such localized regulation could be achieved through controlled drug delivery systems designed to selectively reduce androgen activity at the wound site with minimal systemic impact.

The skin microbiome plays a critical role in regulating wound healing (Patel et al., 2022). In DFUs, up to 90% of bacterial species are reported to be pathogenic, with *Staphylococcus aureus* (*S. aureus*) being the most prevalent (Ding et al., 2022). In our study, castration reshaped the wound microbiota, increasing α-diversity and shifting the bacterial community toward *Escherichia–Shigella (E. coli)*, *Ochrobactrum*, and *Delftia*. Among these, *Escherichia–Shigella* was most strongly associated with improved healing based on SHAP analysis. Functional predictions further revealed that this reshaped microbiota was metabolically more active, particularly in pathways linked to tissue regeneration. These observations align with previous reports showing that successful wound healing is accompanied by an increase in microbial diversity and metabolic activity (Dokoshi et al., 2024). Longitudinal multi-omics cohort studies have consistently shown that non-healing diabetic wounds are characterized by low α-diversity and *Staphylococcus* dominance. Upon healing, both community complexity and functional diversity gradually recover (Bruni et al., 2024; Gardiner et al., 2017; Kalan et al., 2019). *S. aureus* disrupts healing by producing α-toxin, surfactant peptides, and biofilms, which inhibit keratinocyte migration and impair collagen deposition (Roy et al., 2020; Xu et al., 2020).

Interestingly, some conventional opportunistic pathogens such as *E. coli* can play pro-healing roles in specific contexts. Engineered *E. coli* strains that enhance butyrate production have shown the ability to restore mucosal barriers (Lee et al., 2025), and topical application of *Calvatia gigantea* extract has been reported to selectively enrich *Escherichia–Shigella* at the wound site, thereby accelerating healing (Ding et al., 2024). These findings suggest that successful healing depends not merely on eradicating pathogens but on establishing a metabolically complementary and immunologically balanced microbial ecosystem. In the present study, orchiectomy elevated both Chao and Sobs indices, confirming increased α-diversity, and shifted the microbiota toward *Escherichia–Shigella*, *Ochrobactrum*, and *Delftia*–a microbial profile that closely resembled the one previously induced by *Calvatia gigantea* extract (Ding et al., 2024; Harada et al., 2016). Metagenomic inference revealed enrichment of short-chain fatty acid (SCFA) biosynthesis and tryptophan–indole pathways, both of which are implicated in M2 macrophage polarization and enhanced keratinocyte migration, thus expediting tissue regeneration (Morino-Koga et al., 2017). However, we acknowledge that these functional predictions are based on *in silico* analyses and require experimental validation. Future studies could employ germ-free wound models using isolated *Escherichia–Shigella* strain in combination with diverse wound microbiome to assess their effects on diabetic wound healing (Spragge et al., 2023; Xu et al., 2025). Moreover, to gain deeper ecological insight into this microbial community restructuring, future studies could apply network analysis to elucidate inter-taxa relationships.

Collectively, our findings uncover a critical mechanistic link between systemic androgens, the wound microbiome, and the regulation of diabetic wound repair. By demonstrating that androgen deprivation reshapes the microbial community toward a pro-healing state, our study highlights the androgen-microbiome axis as a potential target for intervention (Harada et al., 2016; He et al., 2021; Li et al., 2024). A random forest model identified *Escherichia–Shigella* as the strongest predictor of wound closure, suggesting that specific microbial shifts are key drivers of the accelerated healing process (Jiang et al., 2024). Importantly, this study also highlights sex-based differences in wound healing, which is often overlooked in chronic wound management (Fimmel and Zouboulis, 2005). Given that castration improved healing in our model but is not a clinically feasible option, topical anti-androgens may represent a potential strategy to modulate the wound microbiome while minimizing systemic effects (Hebert et al., 2020).

## 5 Conclusion

This study demonstrates that androgen deprivation through surgical castration significantly accelerates diabetic wound healing, primarily by modulating the local immune response and reshaping the wound microbiome. Castrated diabetic mice exhibited enhanced re-epithelialization, increased extracellular matrix production, and an earlier resolution of inflammation. These benefits were closely linked to increased microbial diversity and enrichment of bacterial taxa such as *Escherichia–Shigella*, which was associated with metabolic pathways that support tissue regeneration, including SCFA and indole biosynthesis. Our findings highlight the critical role of host microbiome interactions in wound healing and suggest that targeting the wound microbiome with topical AR antagonists may represent a promising therapeutic approach. Furthermore, this study underscores the importance of accounting for sex-based biological differences in the management and treatment of chronic wounds.

## Data Availability

The data presented in the study are deposited in the NCBI Sequence Read Archive (SRA), accession number PRJNA1305122, https://www.ncbi.nlm.nih.gov/bioproject/PRJNA1305122.
